# Metformin induces degradation of mTOR protein in breast cancer cells

**DOI:** 10.1002/cam4.896

**Published:** 2016-10-17

**Authors:** Mohamed Alalem, Alpana Ray, Bimal K. Ray

**Affiliations:** ^1^ Department of Veterinary PathobiologyUniversity of MissouriColumbiaMissouri65211

**Keywords:** Breast Cancer, mammalian Target of Rapamycin (mTOR), metformin, mTOR Inhibition, protein degradation

## Abstract

Activation of mTOR is implicated in the development and progression of breast cancer. mTOR inhibition exhibited promising antitumor effects in breast cancer; however, its effect is compromised by several feedback mechanisms. One of such mechanisms is the upregulation of mTOR pathway in breast cancer cells. Despite the established role of mTOR activation in breast cancer, the status of total mTOR protein and its impact on the tumor behavior and response to treatment are poorly understood. Besides, the mechanisms underlying mTOR protein degradation in normal and cancer breast cells are still largely unknown. We and others found that total mTOR protein level is elevated in breast cancer cells compared to their nonmalignant counterparts. We have detected defective proteolysis of mTOR protein in breast cancer cells, which could, at least in part, explain the high level of mTOR protein in these cells. We show that metformin treatment in MCF‐7 breast cancer cells induced degradation of mTOR and sequestration of this protein in a perinuclear region. The decrease in mTOR protein level in these cells correlated positively with a concomitant inhibition of proliferation and migration potentials of these cells. These findings provided a novel mechanism for the metformin action in breast cancer treatment. Understanding the proteolytic mechanism responsible for mTOR level in breast cancer may pave the way for improving the efficacy of breast cancer treatment regimens and mitigating drug resistance as well as providing a basis for potential novel therapeutic modalities for breast cancer.

## Introduction

Mammalian target of rapamycin, mTOR, is a highly conserved serine/threonine kinase, which is ubiquitously expressed in cells to control growth and metabolism 1, 2, 3. This protein is essential for normal development and viability 4 as knockout of mTOR results in embryonic lethality 5, and its ablation in some somatic cells leads to increased apoptosis 6. As a key intermediate in the transmembrane signaling pathway, mTOR integrates various intracellular and extracellular stimuli to regulate many vital cellular processes. Thus, the dysregulation of mTOR pathway is implicated in an increasing number of diseases, including cancer, type 2 diabetes, and neurodegeneration (reviewed in^7^). Hyperactivation of mTOR signaling has been associated with aggressive tumor growth in many cancers 8, including breast cancer 9. The mTOR pathway is implicated not only in tumorigenesis of breast cancer but also in tumor sensitivity to chemotherapy and hormonal treatment. 10. Activated mTOR pathway is known to promote numerous cellular functions consistent with tumor invasiveness such as proliferation, migration, and survival 11.

mTOR is activated in response to nutrients, growth factors, and cellular energy (reviewed in 2, 12). Active mTOR exists in two complexes, mTORC1 and mTORC2, which consist of distinct sets of binding proteins 13. Active mTOR phosphorylates different substrates to regulate distinct cellular functions 14 including protein synthesis, organization of the actin cytoskeleton, membrane traffic, and protein degradation (reviewed in 15). Protein synthesis is a key feature of cancer cells 16 and mTOR regulates protein synthesis through its downstream targets, p70 S6 kinase and eIF4E‐BP 17, 18. Another essential cellular function regulated by mTOR is autophagy 19, which is an intracellular degradation system that delivers cytoplasmic proteins to lysosomes 20.

Involvement of active mTOR pathway in the progression of breast cancer is well established 21, 22. Its inhibition has been shown to sensitize breast cancer cells to the cytotoxic effects of chemotherapy in vitro 23. Rapamycin and its analogs (rapalogs) are highly specific inhibitors of mTOR, and currently, they are being evaluated as anticancer agents in clinical trials. However, toxicity is a limiting factor that precludes the use of high doses of mTOR inhibitors, particularly rapalogs, in combinatorial treatment for breast cancer 24. Moreover, evidence indicates that many human cancers have intrinsic resistance to treatment and the tumors initially sensitive to rapamycin demonstrate acquired resistance and become refractory to the treatment 25. One of the potential mechanisms of the ensuing drug resistance in breast cancer is the upregulation of mTOR pathway, which may involve increased activity or increased levels of total proteins in the mTOR pathway. An earlier report showed that metformin, an antidiabetic agent, exerts antitumor effects via inhibition of mTOR activity 26. Nonetheless, the molecular basis of the beneficial effects of metformin in breast cancer is far from being fully unraveled. Although metformin action on peripheral tissues requires high concentrations, its use is generally tolerable if avoided in patients with contraindications 27. The relatively safe profile of metformin makes it a promising agent for mTOR inhibition in breast cancer, particularly that mTOR inhibitors are usually required in high doses to achieve better antitumor effects.

Total mTOR protein level is high in some cancers, such as colorectal cancer, and it correlates positively with the tumor stage 28, but the status of total mTOR protein and its impact in breast cancer cells are not well delineated. Although several mTOR inhibitors have shown promising antitumor effects 21, there is risk of emergence of drug resistance 29. Notably, feedback upregulation of the mTOR pathway is one of the potential mechanisms of drug resistance in breast cancer. One of the possible mechanisms underlying the upregulation of mTOR pathway is the increased level of total mTOR protein itself. The mechanisms controlling mTOR protein expression and degradation in breast cancer cells are still poorly understood. Autophagy and the ubiquitin‐proteasome system (UPS) are the main intracellular protein degradation pathways in eukaryotes 30. In the UPS, proteins are degraded by the 26S proteasome complex 31. In autophagy, protein degradation is induced by a specific autophagy inducer 32 such as starvation, oxidative stress 33, or proteasome inhibition. In normal cells, constitutive autophagy and the UPS pathways act in parallel to prevent the accumulation of proteins to prevent cells damage, however, the effect of these events in cancer cells are less understood 34. In rapidly proliferating tumor cells, the endoplasmic reticulum sustains stress exceeding the degradative capacity of the proteasome and autophagy systems. As a result, misfolded proteins accumulate in perinuclear aggresomes, which are associated with induction of nonapoptotic cell death 35.

To understand possible mechanisms underlying elevated level of mTOR protein accumulation in breast cancer, we have undertaken this work. Our finding of defective proteolysis of mTOR protein could be potentially exploited for improving the efficacy of breast cancer treatment regimens and mitigating drug resistance as well as providing a basis for potential novel therapeutic modalities for breast cancer.

## Materials and Methods

### Cell lines and reagents

MCF‐10A, MCF‐7, and MDA‐MB‐231 breast cell lines obtained from the ATCC cultured and stored following ATCC protocol of authentication by short terminal repeat analysis. The cells were grown in Dulbecco's modified Eagle's medium (DMEM) containing high glucose (4.5 g/L) (Gibco/Life Technologies , Carlsbad, CA, USA) and supplemented with 7% fetal bovine serum (Harlan Bioproducts for Science, Inc., Indianapolis, IN, USA). Insulin, verapamil, MG132, chymostatin, leupeptin, pepstatin A, metformin, rapamycin, and PP242 purchased from Sigma Chemical Co. (St. Louis, MO, USA).

### Western blot analysis

Cell lysates from both control and treated breast cells were prepared by three rounds of freeze‐thawing and vortexing of cell suspensions in a lysis buffer containing 10 mmol/L 4‐(2‐hydroxyethyl)‐1‐piperazineethanesulfonic acid (HEPES), pH 7.9, 1.5 mmol/L MgCl_2_, 10 mmol/L KCl, 0.5 mmol/L dithiothreitol (DTT), 0.5 mmol/L phenylmethylsulfonyl fluoride (PMSF), 0.5 mg/mL each of leupeptin, antipain, and pepstatin, 0.1 *μ*g/mL chymostatin, 0.3 TIU/mL aprotinin, and 0.5 mg/mL benzamidine. Equal protein amount was fractionated by electrophoresis in sodiumdodecyl sulfate‐polyacrylamide gel (SDS‐PAGE), transferred to PVDF transfer membrane (PerkinElmer, Waltham, MA, USA), stained with Ponceau S solution (Sigma Chemical Co., St. Louis, MO, USA), destained, and immunoblotted with the designated antibodies including *β*‐actin to ensure equal loading. Anti‐mTOR, anti‐pmTOR (Ser‐2448), anti‐pP70S6K (T‐389), anti‐LC3B I, anti‐LC3B II, anti‐*β*‐Actin were purchased from Cell Signaling Technology (Danvers, MA, USA). Anti‐P70‐S6K antibody was purchased from EMD Millipore Corporation(Billerica, MA, USA). Blots were developed with enhanced chemiluminescece reagent (Pierce ECL, Thermo Scientific, Waltham, MA, USA). Band densitometry was measured by AlphaView imaging software, FluorChem Q system, ProteinSimple, and semiquantitative data were normalized for *β*‐actin.

### Protein stability assays

Cycloheximide (CHX) assay was performed by treating the cells with CHX (Sigma Chemical Co.) at 200 *μ*g/mL concentration for various time points, as indicated in Figure legend and the stability of mTOR protein was assessed by western blot (WB).

### Immunocytochemistry

MCF‐7 cells were plated on tissue culture chambers (Lab‐Tek Chamber Slide; Nunc, Inc., St. Louis, MO, USA). Cells were stained with 1 *μ*g/mL Acridine Orange (AO, Hartman‐Leddon Co., Philadelphia, PA, USA), followed by formalin fixation, methanol antigen retrieval, 2% (W/V) fetal bovine serum (FBS) blocking, and anti‐mTOR immunostaining.

### MTT cell proliferation assay

Cells were grown in 24‐well plates to 50–70% confluence and proliferation rate of the cells were determined using a live cell assay kit (CellTiter 96 Non‐Radioactive Cell Proliferation Assay) and following the manufacture's protocol (Promega Corp., Madison, WI, USA). The cells were stained with 3‐(4,5‐dimethylthiazol‐2‐1)‐2,5‐diphenyltetrazolium bromide (MTT, 0.05 mg/mL). Absorbance was recorded at 562 nm using a NanoDrop spectrophotometer (Wilmington, DE, USA).

### Wound healing migration assay

Breast cells were seeded on a flat bottom 24‐well plate, incubated overnight to allow the cells to resume growth. The medium was changed with fresh growth media in 70–80% confluent monolayers and supplemented with insulin and mTOR inhibitors, as described in the Figure legend. Wound was initiated by scratching with a sterile 20‐*μ*L plastic pipette tip. Cell migration, indicated by wound closure, was evaluated by comparing the width of the clear line of cell‐free zone with that of the initial wound using a bright field microscopy. The size of wound was measured at various time points 0, 6, 12, 24, and 48 h.

### Autophagy assay

Cell lysates were fractionated in 4%/8% SDS‐PAGE and immunoblotted for microtubule associated protein 1 light chain 3 isoforms LC3B I and II using antibodies obtained from Cell Signaling Technology. Autophagy was assessed by the relative ratio of LC3BII to LC3BI proteins.

### Statistical analysis

Differences between study groups were analyzed by an one‐way analysis of variance (ANOVA) with a post‐hoc Holm–Sidak method. Results represent the average of three independent experiments (*n = 3; mea*n ± SD, and *P <* *0.05 was considered statistically significant), analyzed by Sigmplot software program 12.3 (Systat Software, Inc., San Jose, CA, www.sigmaplot.com).

## Results

### Level of total mTOR protein is higher in breast cancer cells compared to the noncancerous cells

**Figure 1:**
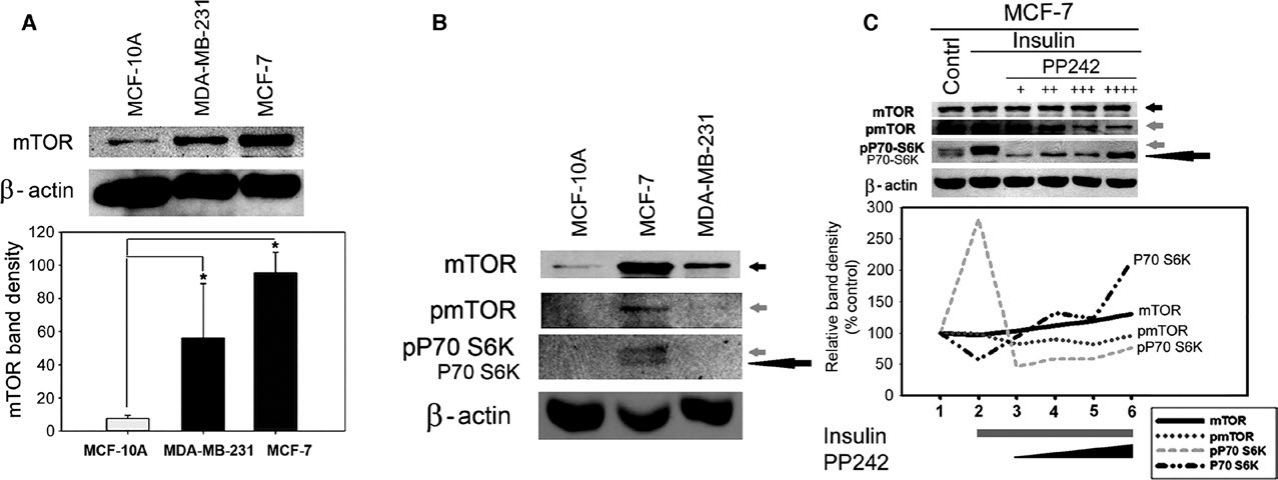
Total mTOR protein level is significantly higher in the breast cancer cells compared to their noncancerous counterparts. (A) Western blot (WB) of 100**** *μ*g of cell lysate protein from MCF‐10A, MCF‐7, and MDA‐MB‐231 cells was performed by immunoblotting with anti‐mTOR and anti‐*β*‐actin antibodies. A densitometric analysis of total mTOR level in the breast cancer cells MCF‐7 and MDA‐MB‐231 was compared to MCF‐10A nontumor cells (*n* = 3, mean ± SD, one‐way analysis of variance (ANOVA) and post‐hoc Holm–Sidak test *P <* *0.05). (B) mTOR activity in MCF‐10A, MCF‐7, and MDA‐MB‐231 cells was determined by the WB analysis of mTOR target proteins using anti‐mTOR, anti‐phospho mTOR, anti‐phospho P70 S6K, anti P70 S6K, and anti‐*β*‐actin antibodies, respectively. (C) MCF‐7 cells treated with insulin (1 *μ*mol/L) for 1 h and different concentrations of mTOR inhibitor PP242 (3, 9, 12, and 15 *μ*mol/L) for 4 h. Cell lysates (100 *μ*g) was immunoblotted for mTOR activity using antibodies as indicated in Panel B. A densitometric analysis of the indicated proteins is shown as a line graph.

To assess the status of total mTOR protein in breast cells, the WB analysis was performed on cancerous and noncancerous breast cell lines. As seen in Figure 1A, total mTOR protein is significantly higher in MCF‐7 and MDA‐MB‐231 breast cancer cells compared to the noncancerous MCF‐10A breast cells. The WB analysis also revealed high levels of phosphorylated mTOR (pmTOR) as well as phosphorylated P70‐S6K (pP70‐S6K), a downstream target of mTOR, in MCF‐7 cells (Fig. 1B). Immunoblotting for pP70‐S6K in the MCF‐7 cells revealed an increase in another band consistent in molecular weight with the total nonphosphorylated form of P70‐S6K protein as shown by the large dark arrow (Fig. 1B). Treatment of MCF‐7 cells with mTOR inhibitor PP242 (Fig. 1C) resulted in an inhibition of mTOR phosphorylation activity in a dose‐dependent manner as evident by the presence of lower levels of pmTOR and pP70‐S6K. However, it also resulted in a concomitant increase in the levels of both mTOR and P70‐S6K, as indicated by the small and large dark arrows, respectively (Fig. 1C). The dose‐dependent effect of PP242 is also represented as line graph (bottom panel, Fig. 1C), which further elucidates above findings. Together, our data suggest that total mTOR protein level is high in breast cancer cells, particularly in the MCF‐7 cells, which correlates with mTOR activity in these cells.

### mTOR protein is more stable in breast cancer cells compared to noncancerous breast cells

**Figure 2:**
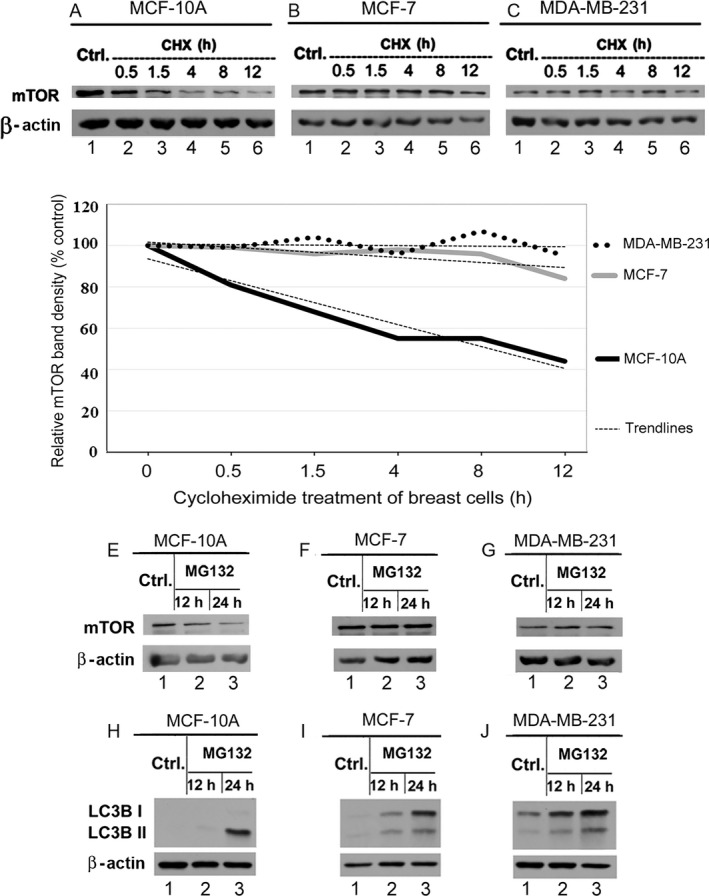
mTOR protein is more stable in breast cancer cells as compared to the noncancerous breast cells. MCF‐10A, MCF‐7, and MDA‐MB‐231 cells were harvested at 0.5, 1.5, 4, 8, and 12 h post 200 *μ*g/mL cycloheximide (CHX) treatment**.** Total cell lysate of MCF‐10A (100 *μ*g in panel A), MCF‐7 (50 *μ*g in panel B), and MDA‐MB‐231(50 *μ*g in panel C) was immunoblotted for mTOR and *β*‐actin. (D) A densitometric analysis of the mTOR protein band in the treatment groups relative to the untreated control groups with a pertinent trend line representation. In a separate experiment, MCF‐10A, MCF‐7, and MDA‐MB‐231 cells were treated with 2 *μ*mol/L of MG132 for several time points (0, 12, 24 h). Total cell lysate of MCF‐10A (100 *μ*g in panels E and H), MCF‐7 (50 *μ*g in panels F and I) and MDA‐MB‐231(50 *μ*g in panels G and J) was immunoblotted for mTOR, LC3B I, LC3B II and *β*‐actin.

The high level of total mTOR protein in the breast cancer cells could be attributed to increased expression and/or reduced degradation of mTOR protein. To investigate the possibility of reduced degradation of mTOR protein in the breast cancer cells, we compared the stability of mTOR protein using CHX) treatment and immunoblotted for mTOR protein (Fig. 2). Our data show that mTOR protein is more stable in MCF‐7 and MD‐MB‐231 breast cancer cells compared to the noncancerous MCF‐10A cells. In MCF‐10A cells, total mTOR protein level declined progressively following CHX treatment (Fig. 2A lanes 2 through 6). However, the level of this protein in both MCF‐7 and MDA‐MB‐231 cells remained relatively unchanged (Fig. 2B and C). These findings (Fig. 2D) suggest that proteolysis of mTOR protein most likely contributes to the lowering the level of this protein in the noncancerous breast cells, but this degradation process is, most likely, less effective in the breast cancer cells.

To assess the nature of proteolysis of mTOR in these cells, we treated the cells with proteasome inhibitor MG132. In MCF‐10A cells, proteasome inhibition resulted in a decrease in the total mTOR protein in a time‐dependent manner (Fig. 2E, lanes 1–3). In contrast, proteasome inhibition caused no significant change in mTOR protein level in both MCF‐7 and MDA‐MB‐231 cells (Fig. 2F and G, respectively). This finding suggested a possibility of proteasome‐dependent mTOR degradation in normal breast epithelial cell, MCF‐10A, but not in the breast carcinoma cells, MCF‐7 and MDA‐MB‐231. Since ubiquitin–proteasome system (UPS) and autophagy are two main proteolytic pathways in eukaryotic cells and these two pathways work in a coordinated and complementary manner so that inhibition of proteasome induces autophagy 30, we examined this possibility. To test, we analyzed the cellular level of LC3B proteins, a family of well‐known autophagy markers 36. As shown in Figure 2H, proteasome inhibition in MCF‐10A cells was associated with increased LC3B II (Fig. 2H, lane 3), which is consistent with activation of autophagy 37. However, in MCF‐7 and MDA‐MB‐231 cells, proteasome inhibition increased both LC3B isoforms with more increase in LC3B I than LC3B II isoform in a time‐dependent manner (Fig. 2I and J, lanes 2 and 3). The accumulation of early intermediates of autophagy, such as LC3B I, likely represents a block in the later stages of autophagy 38. Induction of autophagy marker LC 3BII 39 in MCF‐10A cells following MG132 treatment suggests that proteasome inhibition may have caused induction of autophagy in MCF‐10A cells (Fig. 2H). This event most likely leads to the degradation of mTOR protein in these cells. However, proteasome inhibition in the breast cancer cells did not induce autophagy pathway in breast cancer cells, which had resulted in an increased level of mTOR protein in the cancer cells.

### Metformin treatment of MCF‐7 breast cancer cells decreases the level of total mTOR protein

**Figure 3:**
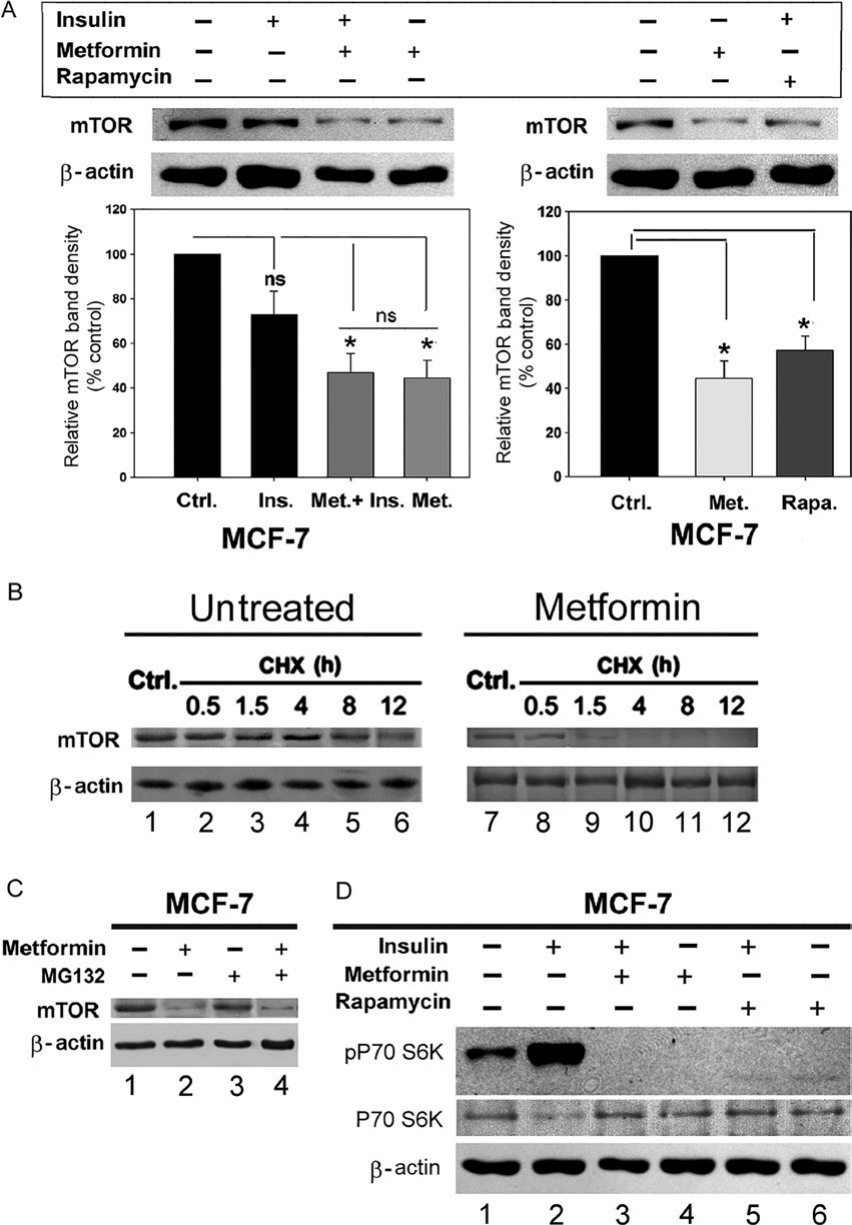
Metformin treatment results in a decrease in the total mTOR protein in breast cancer cells. (A) MCF‐7 cells were treated with insulin (1 *μ*mol/L) for 30 min and/or metformin (75 mmol/L) or rapamycin (100 nmol/L) for 8 h. A quantity of 50 *μ*g of total cell lysate was fractionated and immunoblotted with mTOR and *β*‐actin antibodies. A densitometric analysis of total mTOR level in different treatment conditions, as indicated, is shown as bar graphs (*n* = 3, mean ± SD, one‐way analysis of variance (ANOVA) and post‐hoc Holm–Sidak test *P <* *0.05). (B) MCF‐7 cells were harvested at 0.5, 1.5, 4, 8, and 12 h post 200 *μ*g/mL cycloheximide (CHX) treatment**.** A quantity of 50 *μ*g protein of the total cell lysate was fractionated and immunoblotted for mTOR and *β*‐actin. (C) MCF‐7 cells were treated with metformin (75 mmol/L) and some cells were also treated with MG132 (2 *μ*mol/L) for a total time of 12 h. Total cell lysate (50 *μ*g) was immunoblotted for mTOR and *β*‐actin. (D) MCF‐7 cells were treated with insulin, metformin, and rapamycin as described in panel A. A quantity of 100 *μ*g of cell lysate was fractionated and immunoblotted for phospho‐P70‐S6K, P70‐S6K, and *β*‐actin.

We next assessed whether inhibition of mTOR activity in breast cancer cells impacts mTOR degradation. Treatment of breast cancer cells with metformin and rapamycin, two known mTOR inhibitors, resulted in a significant decrease in the total level of mTOR protein in MCF‐7 cells (Fig. 3A). To assess whether the reduction in mTOR protein after metformin treatment could be due to protein degradation, we measured the mTOR half‐life by CHX experiments in metformin‐treated cells. Our data show higher rate of reduction in mTOR protein in the metformin‐treated cells (Fig. 3B). To test if proteasomal activity is involved in the metformin‐induced mTOR degradation, we treated the MCF‐7 cells with proteasome inhibitor, MG132, with or without metformin treatment. Results, shown in Figure 3C, indicate that MG132 treatment is unable to rescue metformin‐induced mTOR degradation. This finding suggests that mTOR reduction in MCF‐7 is not proteasome‐dependent. Assessment of the effect of metformin and rapamycin treatment on the status of mTOR downstream targets revealed a notable decrease in phospho P70‐S6K (pP70‐S6K). The level of P70‐S6K protein, however, remains mostly unchanged (Fig. 3C).

### Metformin induces a perinuclear sequestration of mTOR protein in breast cancer cells

**Figure 4:**
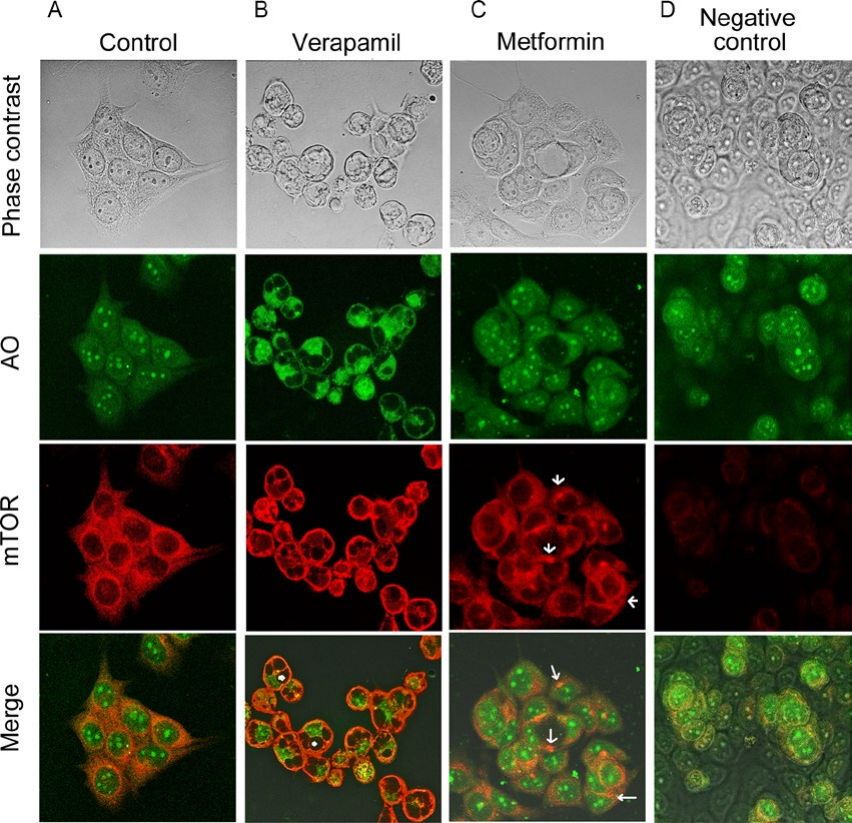
Metformin induced a juxtanuclear aggregation of mTOR protein in the MCF‐7 cells. Confocal immunofluorescence and phase contrast images of MCF‐7 cells (40× magnification) stained with acridine orange (AO) and immunostained for mTOR protein. (A) Untreated (Control) MCF‐7 cells show an even distribution of mTOR protein in the cytoplasm and the absence of focal autophagosomes activity. (B) Arrow heads (in bottom section: Marge) point to vacuoles surrounded by a rim of cytoplasm with relatively intense evenly distributed staining of mTOR as well as Acridine Orange (AO) following treatment with 300 *μ*mol/L of the autophagy‐inducer, verapamil, for 14 h. (C) Metformin (50 mmol/L) treatment for 10 h resulted in a clustering of mTOR proteins in a perinuclear position as indicated by the white arrows in two bottom sections (mTOR and Marge) with no focal increase in autophagosomes in the cytoplasm of MCF‐7 cells. (D) Negative control MCF‐7 cells stained only with AO without immunostaining for mTOR as control for potential background autofluorescence of cellular proteins.

To further verify the involvement of protein degradation in the metformin‐mediated mTOR reduction in MCF‐7 cells, immunocytochemistry for the subcellular localization of mTOR protein was performed. MCF‐7 cells were treated with metformin or verapamil, an autophagy inducer that is known to induce autophagy in vascular smooth muscle cells as well as adenocarcinoma cells 40, 41. The cells were stained with AO for localization of acidic vacuoles in the cytoplasm. Verapamil treatment induced extensive vacuole formation in the cytoplasm of MCF‐7 cells as shown by the arrow heads in Figure 4B, bottom panel, with no apparent effect on mTOR staining in the rim of condensed cytoplasm surrounding the vacuoles. In contrast to verapamil treatment, metformin treatment of MCF‐7 cells did not induce a noticeable vacuolization of the cytoplasm, but it induced accumulation of mTOR protein in the vicinity of the nucleus as shown by the arrows in Figure 4C, bottom two panels, with no focal increase in autophagic activity of AO staining. These findings indicate that metformin treatment induced aggregation of mTOR protein in a perinuclear region consistent with aggresome formation which is known to allow sequestration of misfolded abundant protein molecules and facilitates their clearance by degradation 42.

### The metformin‐induced decrease in mTOR protein level correlates positively with a decrease in the proliferation and migration potentials of MCF‐7 breast cancer cells

**Figure 5:**
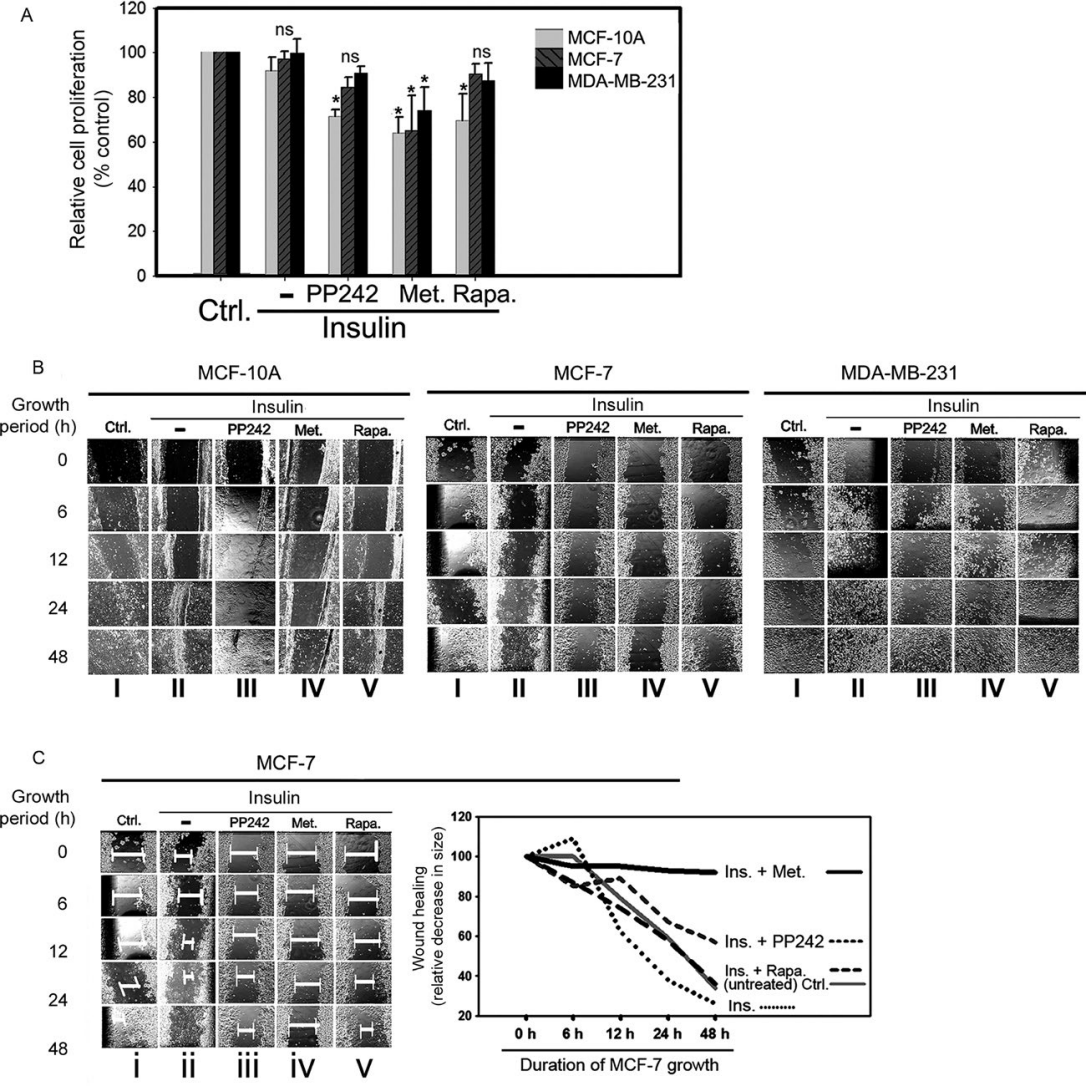
Inhibition of mTOR reduces breast cells’ proliferation and migration. MCF‐10A, MCF‐7, and MDA‐MB‐231 cells were treated with insulin (1 *μ*mol/L) for 12 h in the presence and absence of mTOR inhibitors PP242 (3 *μ*mol/L), metformin (50 mmol/L), and rapamycin (100 nmol/L) for 12 h. (A) A MTT cell proliferation assay for each of the treatment groups was performed as described in Materials and Methods. *P <* *0.05 (B) A wound healing assay was performed following a method as described in Materials and Methods. (C) The distance between the growing edges of migrating cells to bridge the wound was measured under microscope, which is inversely proportional to cells’ potential for migration. The length of thick white lines, which was measured and plotted in the line graph representation, measures the MCF‐7 cell migration in response to mTOR inhibitors.

To examine the impact of metformin‐induced mTOR degradation on the phenotype of breast cancer cells, we compared the effect of various mTOR inhibitors on the proliferation and migration potentials of different breast cells. Metformin treatment of MCF‐7 cells was associated with a marked decrease in the cells proliferation compared to the other mTOR inhibitors (Fig. 5A). These findings were further corroborated by the effect of mTOR inhibitors on the migration of breast cell lines. As shown in the wound healing assay (Fig. 5B), MCF‐7 cells migration decreased profoundly with mTOR inhibition. The migration of MCF‐7 cells was assessed by changes in the wound size in the treatment groups at the designated time points compared to the control groups. The wound size is indicated by the length of thick white line across the wound region (Fig. 5C), which is inversely proportional to the migration potential of the cells. The results show that metformin treatment dramatically inhibited MCF‐7 cells migration (Fig. 5C, track iv compared to the other mTOR inhibitors PP242 and rapamycin (Fig. 5C, tracks iii and v, respectively). The line graph shows that metformin treatment in particular dramatically inhibited MCF‐7 cells migration.

## Discussion

The findings of this work emphasized results of previous research about beneficial role of mTOR inhibition in breast cancer. This study showed an evidence that metformin and rapamycin resulted in a decrease in the overall level of mTOR protein in MCF‐7 breast cancer cells in addition to the inhibition of mTOR activation. Compared to other mTOR inhibitors, such as rapamycin and PP242, metformin treatment exerted more inhibitory effect on proliferation and migration of breast cancer cells. Furthermore, metformin elicited less rebound upregulation of total proteins in the mTOR pathway (namely, P70 S6K, as seen in Fig. 3C) in breast cancer cells, which potentially imposes a lesser risk of emergence of drug resistance to mTOR inhibition in breast cancer treatment regimens.

This study revealed that total mTOR protein is higher in the breast cancer cells compared to the noncancerous cells, which correlated positively with the level of mTOR activity (Fig. 1). Therefore, high mTOR protein could be potentially involved in promoting the cancerous phenotype of breast cancer cells. This hypothesis is substantiated by the relatively strong correlation between the decreased total mTOR protein induced by metformin (Fig. 3) and the resultant inhibition of proliferation and migration of breast cancer cells (Fig. 5).

The decreased mTOR protein degradation is one of the potential causes underlying the high mTOR protein level in breast cancer cells. Our findings revealed that mTOR protein is degraded more rapidly in the noncancerous breast cells compared to the breast cancer cells (Fig. 2). These findings suggest that the rate of mTOR degradation in breast cancer cells is, most likely, lower compared to that in the noncancerous cells. Such a difference between normal and cancer breast cells could be exploited to open a new avenue for novel antitumor agents by targeting these mechanisms preferentially in the breast cancer cells. Our data in Figures 3 and 4 provided evidence that metformin may be able to induce mTOR degradation in breast cancer cells by triggering aggresome formation.

This study revealed an increase in the LC3B I more than LC3B II isoform in breast cancer cells upon proteasome inhibition (Fig. 2), which suggests that these cells are likely to initiate autophagy, yet unable to finish the conversion process. These findings together could, at least in part, explain the high mTOR level in the breast cancer cells and low mTOR level in the noncancerous cells.

Treatment of MCF‐7 cells with a known autophagy inducer, verapamil 40, 41, induced vacuolization of the cytoplasm consistent with autophagosome formation, but metformin treatment; however, did not induce such vacuolization. Instead, metformin treatment induced accumulation of mTOR protein in a perinuclear aggresome. Accumulating proteins in cells are generally transported toward the microtubule organizing center, where they are sequestered into a single large perinuclear aggresome 43. Aggresome formation allows accumulated proteins to be sequestered in aggresome and facilitates their clearance by autophage 42 Our results show that metformin induced sequestration of mTOR in perinuclear aggregation (Fig. 4). Metformin treatment also resulted in increased degradation of cytoskeletal proteins, which could explain decreased viability and proliferation of MCF‐7 cells after metformin treatment. Our finding of growth regulation of metformin‐treated breast cancer cells (Fig. 5) is consistent with a previous finding which showed metformin‐induced inhibition of MCF‐7 cell proliferation in an AMPK‐dependent manner 44. Since activation of AMPK causes inhibition of mTOR 45, 46, 47, our finding raises the possibility that AMPK–mTOR signaling event might also be involved in breast cancer cell growth inhibition. Furthermore, metformin induced degradation of mTOR (Fig. 3) plays an important role in triggering cell growth inhibition. These findings provide a novel mechanism involving the mode of action of metformin in breast cancer cells, could be utilized in improving the efficacy of breast cancer treatment, and counteracting emergence of resistance in breast cancer cells to the treatment modalities.

## Conflicts of Interest

No potential conflicts of interest were disclosed by the authors.
